# Antioxidant PDA-PEG nanoparticles alleviate early osteoarthritis by inhibiting osteoclastogenesis and angiogenesis in subchondral bone

**DOI:** 10.1186/s12951-022-01697-y

**Published:** 2022-11-16

**Authors:** Zhikai Wu, Kai Yuan, Qian Zhang, Jiong Jiong Guo, Huilin Yang, Feng Zhou

**Affiliations:** 1 Department of Orthopaedic Surgery, The First Affiliated Hospital of Soochow University, No. 899 Ping Hai Road, Suzhou, Jiangsu China; 2 Orthopaedic Institute, Soochow University, Suzhou, Jiangsu China; 3 Shanghai Key Laboratory of Orthopaedic Implants, Department of Orthopaedic Surgery, Shanghai Ninth People’s Hospital, Shanghai Jiao Tong University School of Medicine, Shanghai, China

**Keywords:** PDA-PEG NPs, Osteoarthritis, Osteoclastogenesis, Angiogenesis, ROS, PDGF-BB

## Abstract

Accumulating evidence suggests that osteoclastogenesis and angiogenesis in subchondral bone are critical destructive factors in the initiation and progression of osteoarthritis (OA). Herein, methoxypolyethylene glycol amine (mPEG-NH_2_) modified polydopamine nanoparticles (PDA-PEG NPs) were synthesized for treating early OA. The cytotoxicity and reactive oxygen species (ROS) scavenging ability of PDA-PEG NPs were evaluated. The effects of PDA-PEG NPs on osteoclast differentiation and vessel formation were then evaluated. Further, PDA-PEG NPs were administrated to anterior cruciate ligament transection (ACLT)-induced OA mice. Results demonstrated that PDA-PEG NPs had low toxicity both in vitro and in vivo. PDA-PEG NPs could inhibit osteoclastogenesis via regulating nuclear factor kappa B (NF-κB) and mitogen-activated protein kinase (MAPK) signaling pathways. Moreover, PDA-PEG NPs suppressed osteoclast-related angiogenesis via down-regulating platelet-derived growth factor-BB (PDGF-BB). In vivo, PDA-PEG NPs inhibited subchondral bone resorption and angiogenesis, further rescuing cartilage degradation in OA mice. In conclusion, we demonstrated that PDA-PEG NPs deployment could be a potential therapy for OA.

**Graphical Abstract:**

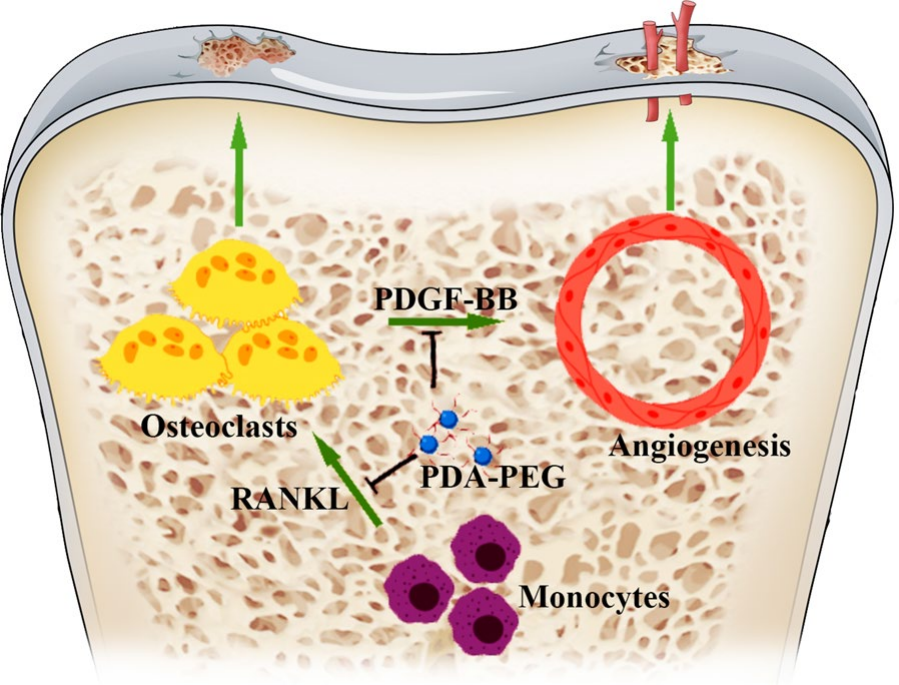

**Supplementary Information:**

The online version contains supplementary material available at 10.1186/s12951-022-01697-y.

## Introduction

Osteoarthritis (OA) is characterized by progressive cartilage degradation, subchondral bone remodeling, and osteophyte formation [1, 2]. As the most prevalent form of arthritis, OA affects millions of people, causing disability due to pain [3]. OA could lead to limited joint mobility, seriously affecting the quality of life [4]. Conservative treatments for OA such as taking non-steroidal anti-inflammatory drugs (NSAIDs) could temporarily relieve pain [5]. However, the deployment of NSAIDs is restricted due to their side effects such as peptic ulcers [6]. Joint replacement is considered a definitive treatment for advanced OA [7]. Considering the significant surgical risk and high financial costs, it is necessary to further understand OA and find innovative treatments.

OA is a whole-joint disease that typically affects all of the components in the joint including cartilage, subchondral bone, and synovium [8]. Aetiology of OA is multifactorial and remains to be further investigated [9]. The subchondral bone and articular cartilage are closely linked and combined, forming a special functional unit the namely osteochondral unit. During the progression of OA, the osteochondral unit would undergo specific alterations including composition, function, structure, and cellular activities [10]. Recently, increasing evidence has demonstrated that osteoclast activity and bone remodeling rate were increased in the early stage of OA [11]. As a type of bone cell, osteoclast secretes acid and collagenase for disassembling hydrated proteins and minerals, which is known as osteolysis [12]. Increased osteoclast activity could disturb the equilibrium between bone formation and resorption, leading to a marked subchondral bone reduction [13]. Moreover, the imbalance between bone formation and resorption could cause abnormal subchondral bone reconstruction, further promoting OA progression [14]. Abnormal vascularization has been found to originate from subchondral bone and stretch to the tide mark in the early stage of OA [15]. Angiogenesis in subchondral bone is regulated by several secretory factors from different bone cells [16]. In particular, pre-osteoclasts could secrete platelet-derived growth factor-BB (PDGF-BB), which is an important pro-angiogenic factor that promotes vessel formation [17]. Moreover, it was demonstrated that the inhibition of angiogenesis in subchondral bone might attenuate OA progression [18]. Thus, suppressing osteoclast formation and the subsequent angiogenesis in subchondral bone might be a potential intervention strategy for treating OA.

Reactive oxygen species (ROS) are important damaging factors during OA progression [19]. Under physiological conditions, ROS, which include free radicals and reactive molecules such as superoxide anion (O2·^−^), hydroxyl radical (HO·), and hydrogen peroxide (H_2_O_2_), are essential for cell signals transmission, energetic cycling, and extracellular matrix metabolism [20]. However, excessive ROS amounts will break normal signaling and homeostasis [21]. It has been demonstrated that ROS are important components that regulate osteoclasts differentiation [22]. Therefore, inhibiting ROS might be a therapeutic potential for osteoclast-related diseases including OA.

The therapeutic potentials of nanoparticles (NPs) for ROS relevant diseases have attracted widespread attention [23]. As a type of naturally-occurring biopolymers, polydopamine (PDA) has active surface functionality, which is appropriate for loading responsive motifs [24]. PDA based materials have shown abundant reductive properties [25, 26]. PDA NPs could scavenge ROS remarkably, hence inhibiting the acute inflammation-induced injury [27, 28]. Moreover, systemic investigations showed the high biocompatibility and low systemic toxicity of PDA NPs both in vitro and in vivo [29]. Polyethylene glycol and its derivatives are frequently used for surface modification in the field of materials science [30]. In this study, methoxypolyethylene glycol amine (mPEG-NH_2_) modified PDA (PDA-PEG) NPs were synthesized. We analyzed the biosecurity and antioxidative effects of PDA-PEG NPs. The effects of PDA-PEG NPs on osteoclast differentiation and angiogenesis were assessed. Additionally, PDA-PEG NPs were deployed to treat anterior cruciate ligament transection (ACLT)-induced OA mice. We aimed to provide a novel therapy for OA.

## Results

### Characterization of PDA-PEG NPs

**Fig. 1:**
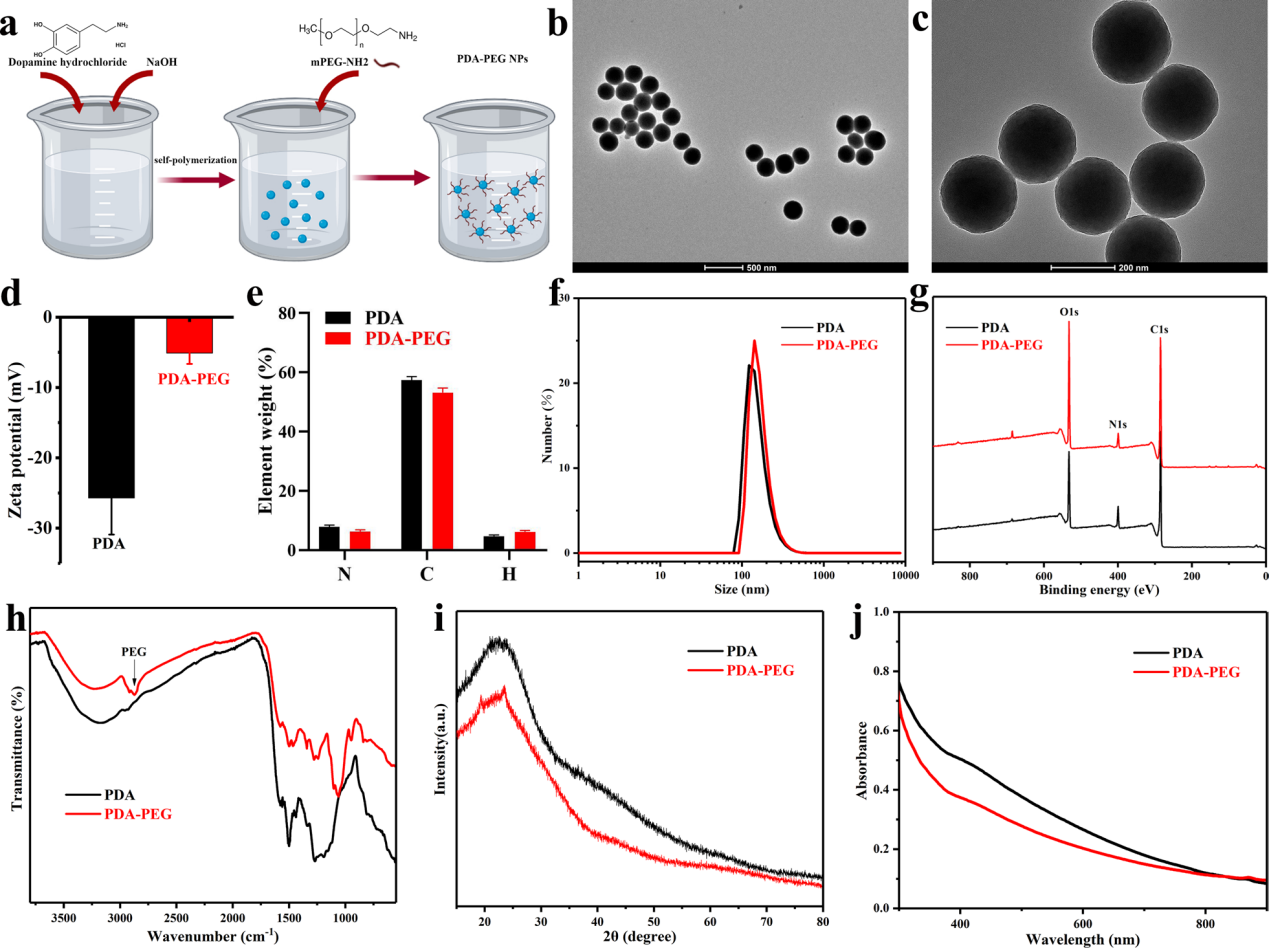
Synthesis and characterization of PDA-PEG NPs. **a** Schematic illustration of the synthesis of PDA-PEG NPs. TEM images of PDA-PEG NPs at **b** low magnification and **c** high magnification. **d** Zeta potentials analysis of PDA and PDA-PEG NPs. **e** Element weight of PDA and PDA-PEG NPs including N, C, and H. **f** DLS analysis, **g** XPS, **h** FT-IR spectra, **i** XRD patterns, and **j** UV–Vis-NIR spectrum of PDA and PDA-PEG NPs. n = 3

The synthesis process of PDA-PEG NPs was shown in Fig. 1a. PDA-PEG NPs were synthesized with a uniform spherical morphology photographed with a transmission electron microscope (TEM) (Fig. 1b, c). As shown in Fig. 1d, the zeta potential values of PDA and PDA-PEG NPs were − 25.7 ± 5.2 and − 5.1 ± 1.53 mV, respectively. Element analysis showed the element weight of PDA and PDA-PEG NPs including nitrogen (N), carbon (C), and hydrogen (H) (Fig. 1e). Dynamic light scattering (DLS) results showed that the mean size of PDA-PEG NPs was ~ 200 nm (Fig. 1f). X-ray photoelectron spectrometer (XPS) analysis illustrated composition of the NPs. Results indicated that PDA and PDA-PEG NPs showed the same peak components for O1s, N1s, and C1s (Fig. 1g). In Fourier transform infrared (FTIR) spectra analysis, peak at about 2900 cm^− 1^ represented the C-H stretching vibration, which suggested the successful modification of PEG on PDA NPs [31]. PDA and PDA-PEG NPs were then detected by X-ray diffraction (XRD) and UV-Vis-NIR spectrum (Fig. 1i, j). The obtained XRD patterns of PDA and PDA-PEG were consistent with those of previous studies [32, 33]. These data indicated the successful synthesis of PDA NPs and the successful modification of PEG on PDA NPs.

### PDA-PEG NPs inhibit ROS in acellular/intracellular environments

**Fig. 2:**
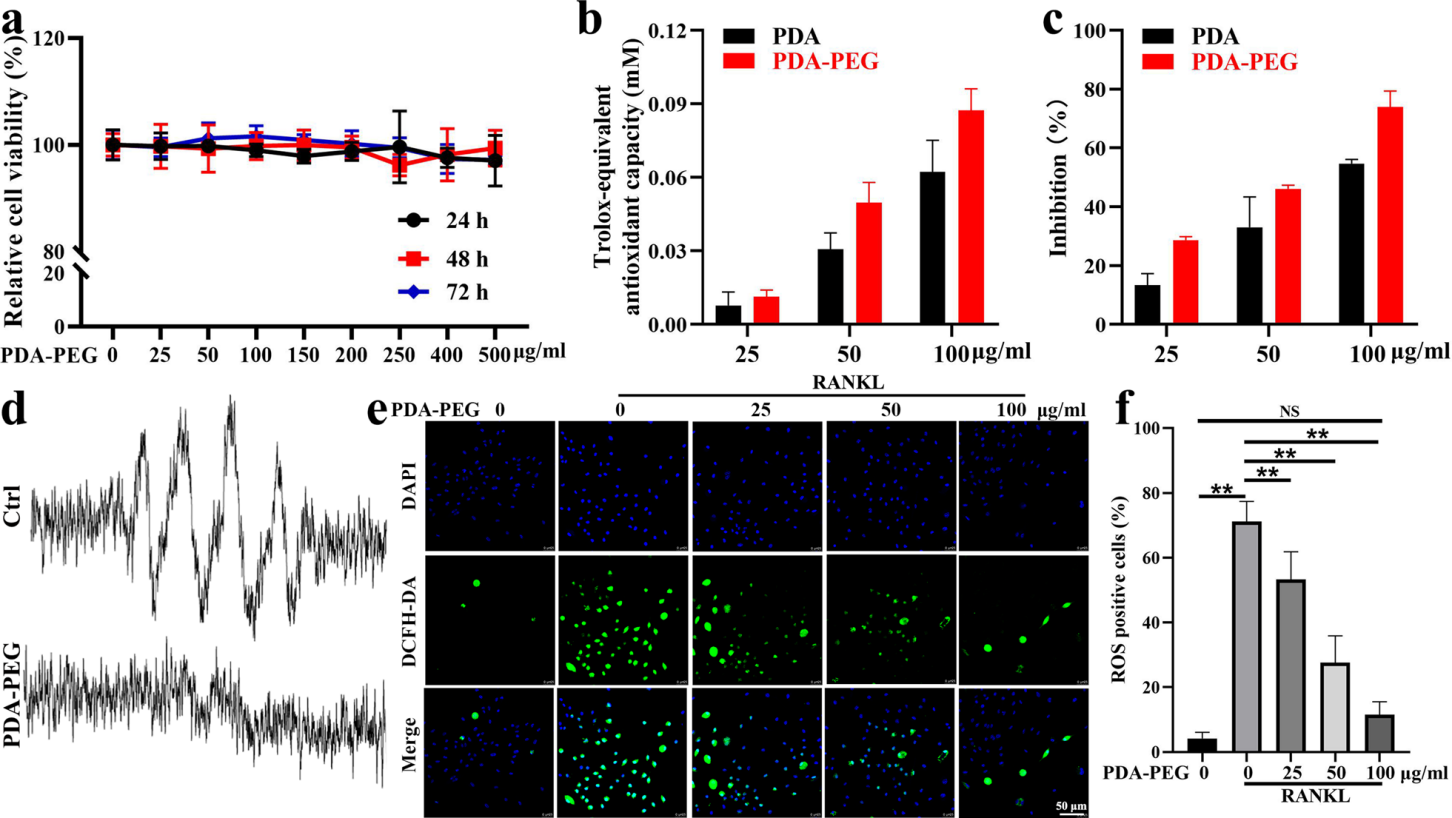
ROS scavenging ability of PDA-PEG NPs. **a** Cytotoxicity of PDA-PEG NPs assessed by CCK-8. Free radical scavenging activities of PDA and PDA-PEG NPs were evaluated with **b** ABTS and **c** DPPH assay. **d** O2·^−^ was determined with ESR for detecting the ROS scavenging ability of PDA-PEG NPs in an acellular environment. **e** Intracellular ROS detected with a DCFH-DA probe, ×630 magnification. **f** Quantitative analysis of ROS-positive cells. n = 3. Symbol “**” denotes p < 0.01, NS indicates not significant

First, the cytotoxicity of PDA-PEG NPs was analyzed with a CCK-8 assay. Results demonstrated that PDA-PEG NPs had minimal cytotoxicity even at high concentrations of 500 µg/ml for 72 h (Fig. 2a). 2,2′-azino-bis(3-ethylbenzothiazoline-6-sulfonic acid) (ABTS) and 2,2-diphenyl-1-picrylhydrazyl (DPPH) were used to assess the antioxidant activities of NPs in water and organic solvent, respectively. Results showed that both PDA and PDA-PEG NPs could scavenge ROS, especially at high concentrations. Moreover, the antioxidant activities of PDA NPs were enhanced to some degree with the modification of mPEG-NH_2_ (Fig. 2b, c). Further, electron spin resonance (ESR) assay was performed to validate the ROS scavenging ability of PDA-PEG NPs in an acellular environment. Results suggested that PDA-PEG NPs at 100 µg/ml could effectively reduce O2·^−^ peak amplitude in contrast to the control group (Fig. 2d). In bone marrow-derived monocytes (BMMs) treated with receptor activator of nuclear factor-kappa B ligand (RANKL), intracellular ROS levels were increased compared to the control group. Nevertheless, PDA-PEG NPs significantly inhibited ROS generation in a dose-dependent manner (Fig. 2e, f). These results suggested that PDA-PEG NPs were able to effectively suppress ROS both in the acellular and intracellular environments.

### PDA-PEG NPs suppress osteoclastogenesis in vitro

**Fig. 3:**
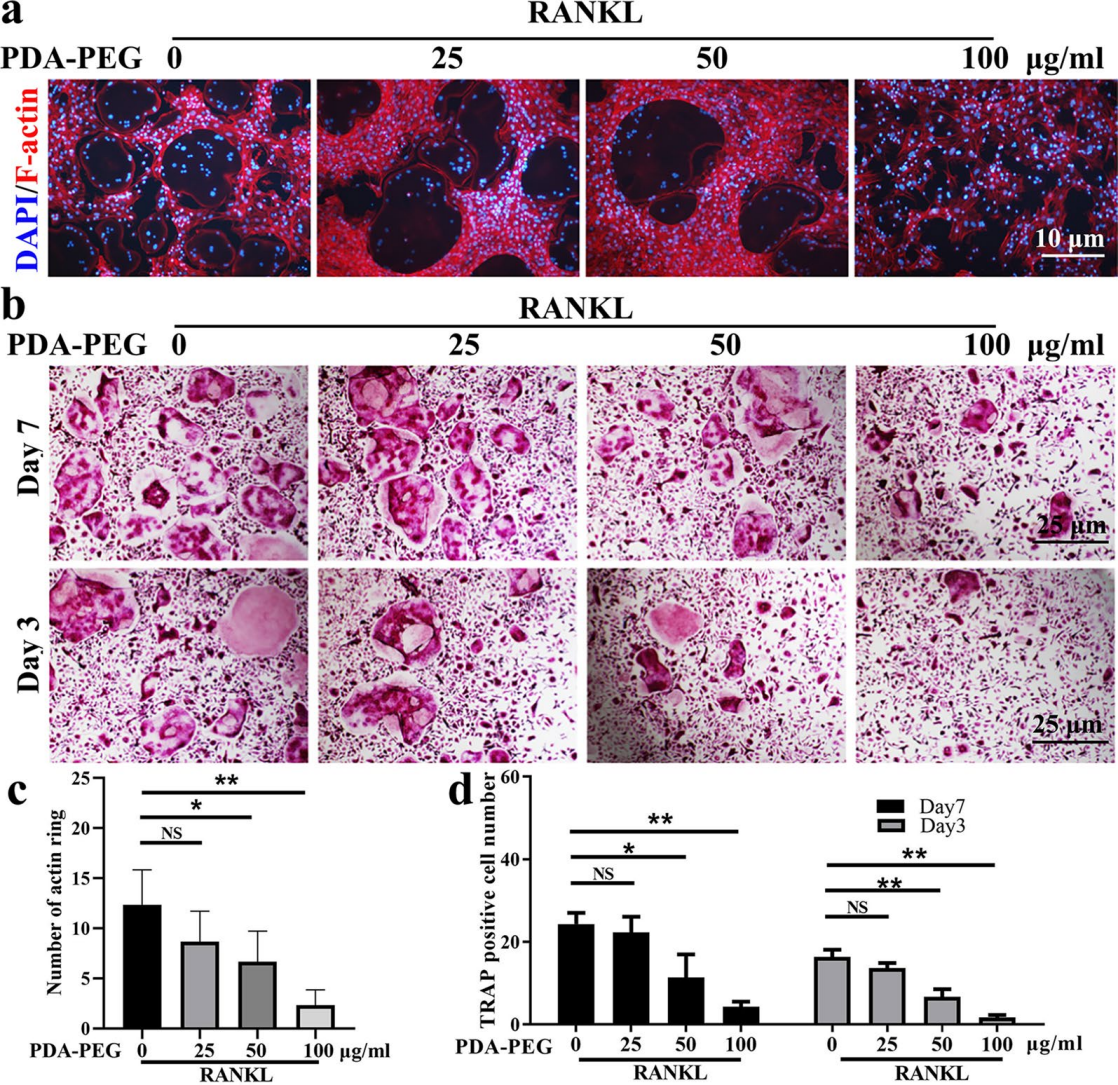
PDA-PEG NPs inhibits osteoclast formation. **a** F-actin ring staining of BMMs treated with RANKL and various concentration of PDA-PEG NPs, ×200 magnification. **b** TRAP staining of BMMs on the 3rd and 7th days, ×100 magnification. **c** Quantification of actin ring per field. **d** Quantification of TRAP-positive cells per field. n = 3. Symbols “*” denotes p < 0.05, “**” denotes p < 0.01, NS indicates not significant

Effects of PDA-PEG NPs on osteoclast formation were assessed using actin ring and tartrate-resistant acid phosphatase (TRAP) staining. BMMs were treated with macrophage-colony stimulating factor (M-CSF) and RANKL to induce osteoclast differentiation. During the induction, varying concentrations (0, 25, 50, and 100 µg/ml) of PDA-PEG NPs were added. The number of acting rings was reduced with the stimulation of PDA-PEG NPs (Fig. 3a, c). Additionally, numerous TRAP-positive multinucleated osteoclasts formed with the treatment of M-CSF and RANKL, while PDA-PEG NPs suppressed osteoclast formation dose-dependently on both the 3rd and 7th days (Fig. 3b, d). These data implied that PDA-PEG NPs could suppress osteoclast formation in vitro.

### PDA-PEG NPs inhibit osteoclastogenesis-related genes and proteins

**Fig. 4:**
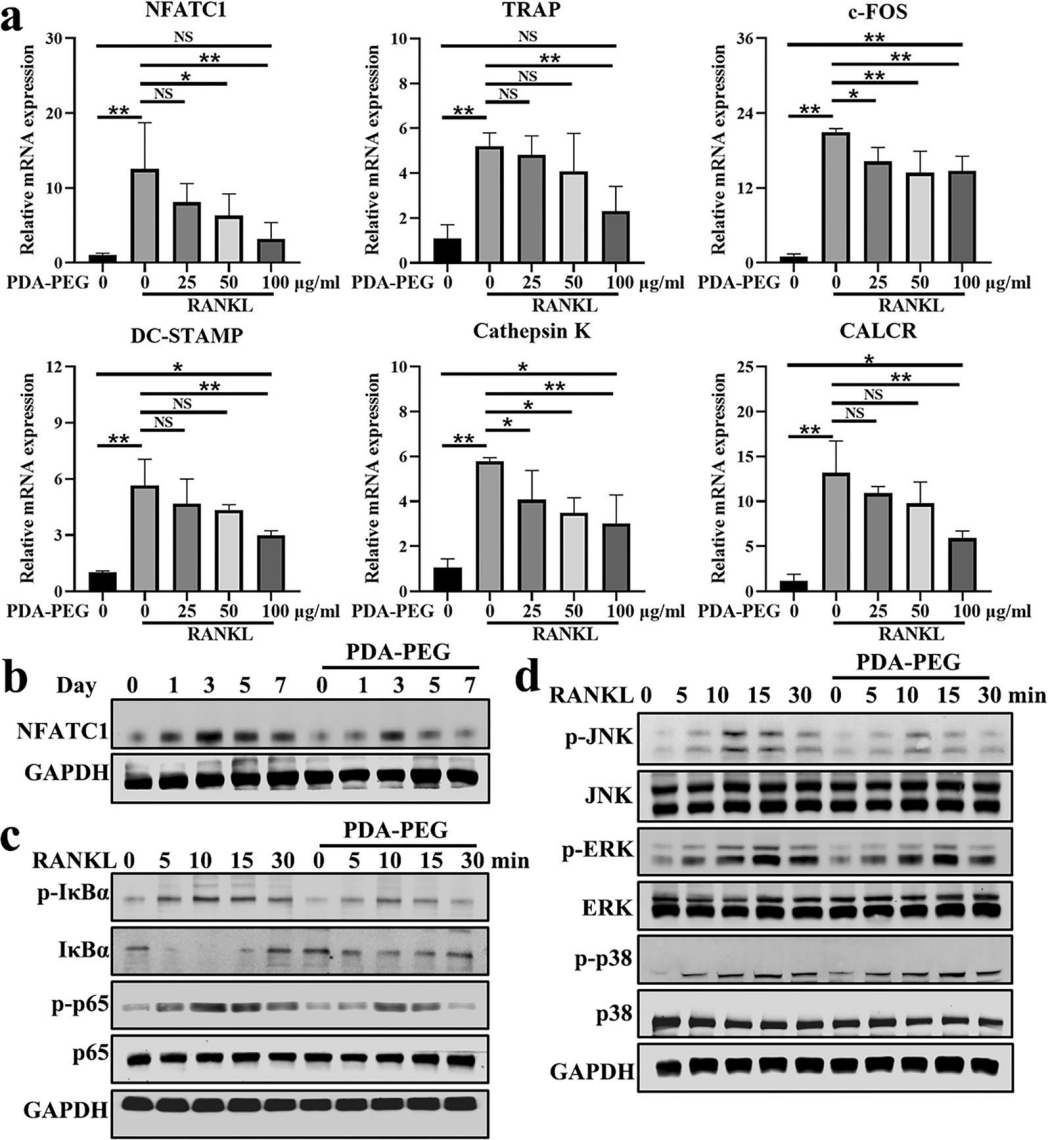
PDA-PEG NPs regulates osteoclastogenesis-related genes and proteins. **a** Expression of osteoclast-related genes including NFATC1, TRAP, c-FOS, DC-STAMP, cathepsin K, and CALCR. n = 3. **b** Expression of NFATC1 protein assessed by western blotting. **c** Effects of PDA-PEG NPs on NF-κB pathway proteins including p-IκBα/IκBα, p‐p65/p65. **d** MAPK signaling pathway proteins including p-ERK/ERK, p-JNK/JNK, and p-p38/p38 were evaluated. Symbol “*” denotes p < 0.05, “**” denotes p < 0.01, NS indicates not significant

Several molecules are involved in osteoclast differentiation [34]. The expressions of osteoclastogenesis-related genes which included nuclear factor of activated T-cells 1 (NFATC1), TRAP, c-FOS, dendritic cell-specific transmembrane protein (DC-STAMP), cathepsin K, and calcitonin receptor (CALCR) were analyzed using real-time quantitative reverse transcription polymerase chain reaction (qRT-PCR). Results demonstrated that the expression of the genes encoding osteoclast formation was significantly increased with the stimulation of RANKL. Nevertheless, PDA-PEG NPs dose-dependently suppressed their expression (Fig. 4a). Moreover, we utilized western blotting to assess the expression of NFATC1 protein, which is an important transcription factor for osteoclastogenesis [35]. Results showed that 100 µg/ml of PDA-PEG NPs could inhibit NFATC1 expression at various time points (Fig. 4b). These data suggested that PDA-PEG NPs suppressed osteoclast-related genes and proteins in vitro.

Nuclear factor kappa B (NF-κB) signaling pathway was shown to be closely related to osteoclastogenesis [36]. As shown in Fig. 4c, western blotting results demonstrated that RANKL stimulation significantly increased expression of p-IκBα and promoted degradation of IκBα. However, PDA-PEG NPs treatment inhibited phosphorylation of IκBα while upregulated IκBα expression. In the downstream of NF-κB, PDA-PEG NPs treatment suppressed phosphorylated p65 compared to RANKL-induced BMMs. Mitogen-activated protein kinase (MAPK) signaling pathway, mainly composed of ERK, JNK, and p38, is crucial for activating osteoclast formation and function [22]. Figure 4d showed that the deployment of RANKL promoted phosphorylation of JNK, ERK, and p38, while p-JNK protein was decreased after PDA-PEG NPs treatment. These results implied that PDA-PEG NPs might inhibit osteoclast formation via regulating NF-κB/JNK pathway.

### PDA-PEG NPs attenuate H_2_O_2_-induced osteoclast formation

**Fig. 5:**
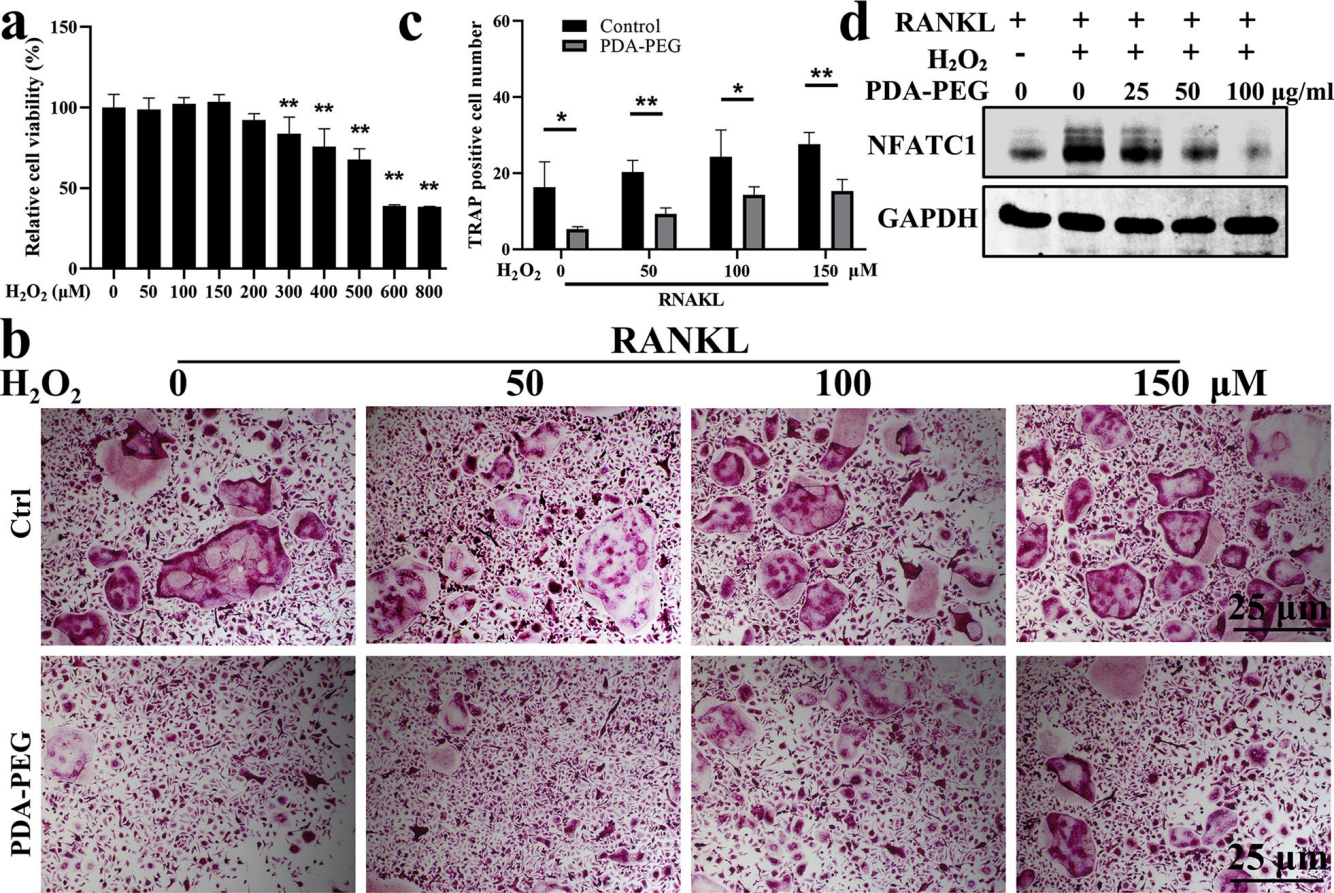
PDA-PEG NPs inhibits H_2_O_2_-induced osteoclast differentiation. **a** BMMs treated with various concentrations of H_2_O_2_ then assessed by CCK-8. **b** TRAP staining of BMMs treated with exogenous H_2_O_2_ and PDA-PEG NPs on the 3rd day, ×100 magnification. **c** Quantification of TRAP-positive cells per field. **d** Effect of PDA-PEG NPs on NFATC1 expression determined by western blotting. n = 3. Symbol “*” denotes p < 0.05, “**” denotes p < 0.01

ROS are important components for regulating osteoclasts differentiation [22]. In this study, exogenous H_2_O_2_ was deployed during osteoclastogenesis. We first determined the cytotoxic effects of H_2_O_2_ on BMMs. Results showed that concentrations of H_2_O_2_ higher than 200 µM could inhibit cell proliferation (Fig. 5a). Hence, 50, 100, and 150 µM H_2_O_2_ was administrated during osteoclast formation. Results demonstrated that exogenous administration of H_2_O_2_ could promote osteoclastogenesis. However, PDA-PEG NPs treatment at 100 µg/ml could attenuate the H_2_O_2_-induced osteoclast formation (Fig. 5b, c). Western blotting results confirmed that exogenous H_2_O_2_ could promote expression of NFATC1 during osteoclastogenesis, which was inhibited by PDA-PEG NPs in a dose-dependent manner (Fig. 5d). These data demonstrated that PDA-PEG NPs could attenuate H_2_O_2_-induced osteoclastogenesis.

### PDA-PEG NPs modulate osteoclast-related angiogenesis

**Fig. 6:**
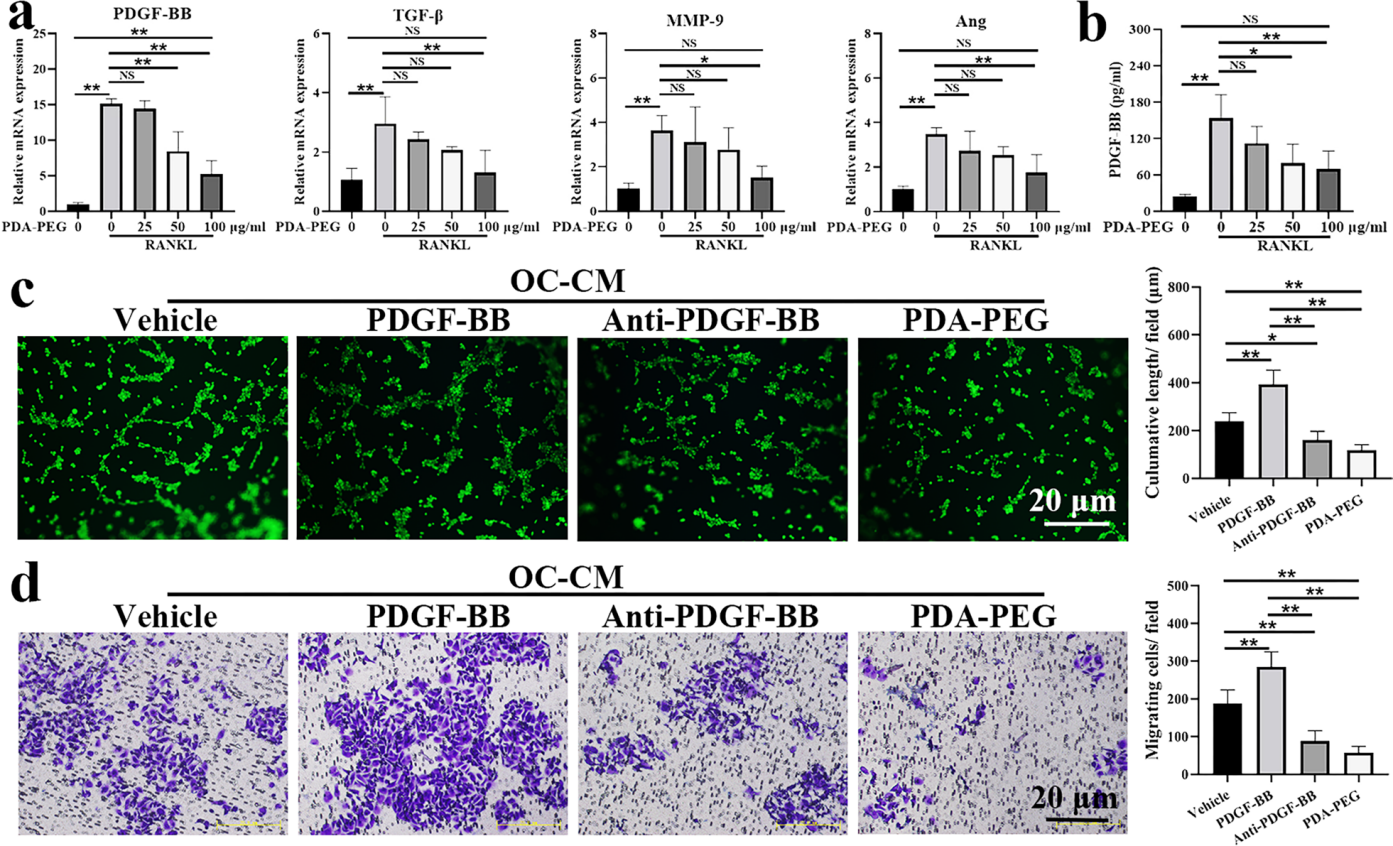
PDA-PEG NPs inhibits osteoclast-related angiogenesis. **a** Expressions of angiogenesis-related genes including PDGF-BB, TGF-β, MMP-9, and Ang were assessed by qRT-PCR. **b** Quantitative analysis of PDGF-BB determined by ELISA. **c** Effect of PDA-PEG NPs on tube formation, ×100 magnification. **d** Effect of PDA-PEG NPs on cell migration, ×100 magnification. n = 3. Symbol “*” denotes p < 0.05, “**” denotes p < 0.01, NS indicates not significant

It has been shown that osteoclast could promote angiogenesis via secreting proangiogenic factors [17]. We evaluated expression of the angiogenesis-related genes including PDGF-BB, transforming growth factor-β (TGF-β), matrix metalloproteinse-9 (MMP-9), and angiopoietin (Ang) during osteoclast differentiation. Our results show that the stimulation of RANKL could promote expressions of PDGF-BB, TGF-β, MMP-9, and Ang, which were inhibited by PDA-PEG NPs, especially at a high concentration (Fig. 6a). As the fold change in PDGF-BB was most significant, we further quantitated PDGF-BB in osteoclast-conditioned medium (OC-CM) utilizing the enzyme-linked immunosorbent assay (ELISA) kit. Results demonstrated that the concentration of PDGF-BB in OC-CM was 153.44 ± 38.88 pg/ml, which was inhibited with the deployment of PDA-PEG NPs dose-dependently (Fig. 6b). The tube formation assay was administrated to visually evaluate the effects of OC-CM on angiogenesis. As shown in Fig. 6c, the length of the tube in OC-CM treated human umbilical vein endothelial cells (HUVECs) was 238.10 ± 36.83 μm, which was enhanced with exogenous treatment of PDGF-BB. While, the length of the tube was reduced with anti-PDGF-BB antibody or PDA-PEG NPs treatment. Results of the migration assay demonstrated that PDGF-BB could promote migration of HUVECs, meanwhile the migration was suppressed with the deployment of anti-PDGF-BB antibody or PDA-PEG NPs (Fig. 6d). These results showed that PDA-PEG NPs could inhibit OC-CM induced angiogenesis via modulating the key proangiogenic factor PDGF-BB.

### PDA-PEG NPs suppress subchondral bone resorption and angiogenesis in OA mice

**Fig. 7:**
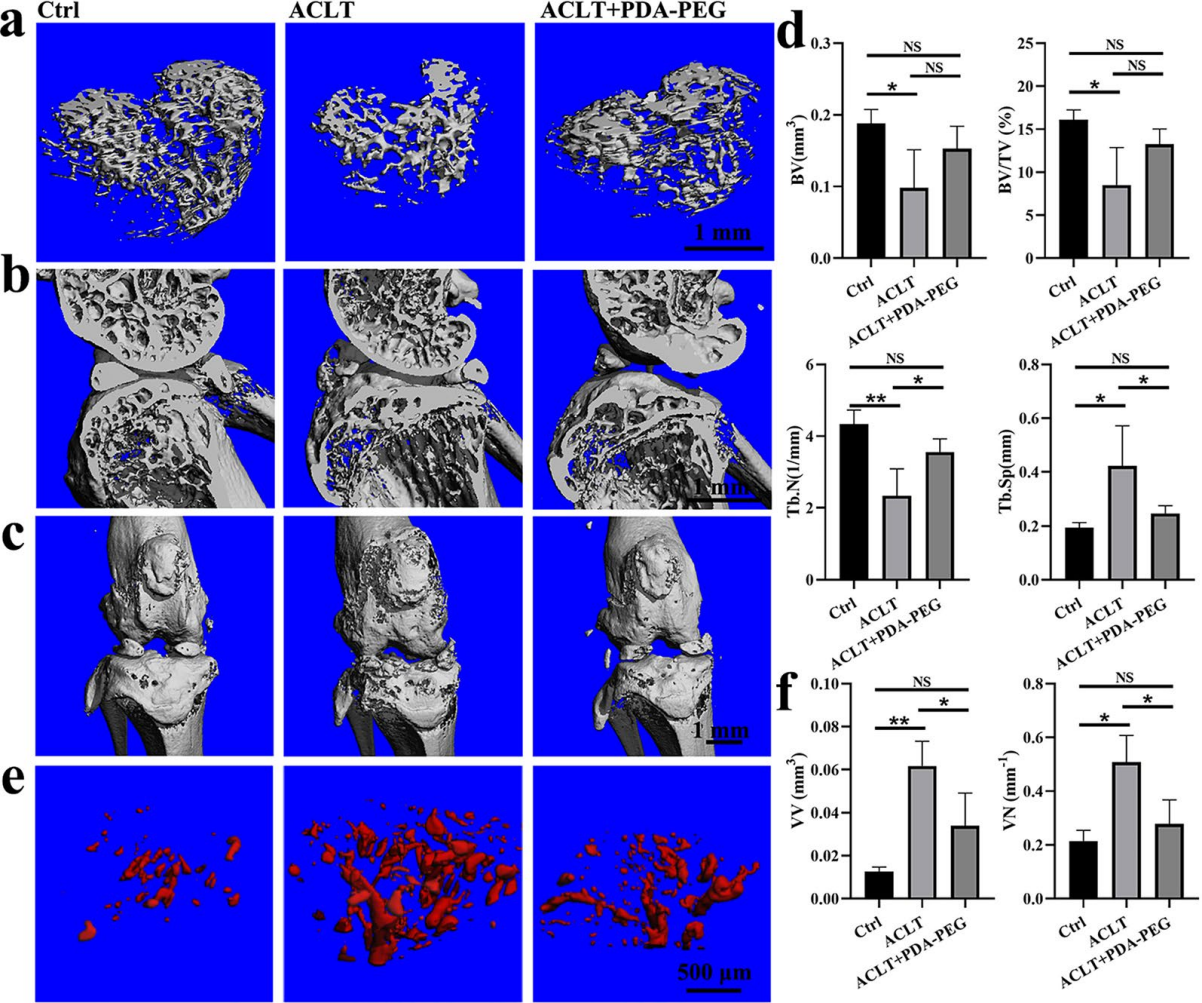
PDA-PEG NPs suppresses bone resorption induced by ACLT. **a** Three-dimensional reconstruction of the subchondral bone. **b** Sagittal views and of the medial compartment of the knee. **c** Front views of the knee joints. **d** Quantification of micro-CT parameters including BV, BV/TV, Tb.N, and Tb.Sp. **e** Reconstruction of subchondral blood vessels based on microangiography. **f** Quantification of VV and VN of the blood vessels. n = 3. Symbol “*” denotes p < 0.05, “**” denotes p < 0.01, NS indicates not significant

To investigate the effects of PDA-PEG NPs on osteoclast and blood vessel formation in vivo, we administered PDA-PEG NPs intraperitoneally in ACLT-induced OA mice. As indicated in Fig. 7a and b, three-dimensional reconstruction of the subchondral bone and sagittal views of the medial compartment of knee showed that bone resorption was increased after ACLT induction. Nonetheless, the destruction was reduced by PDA-PEG NPs, indicating that osteolysis was inhibited after PDA-PEG NPs treatment. Moreover, the bone lesions induced by ACLT was reduced by PDA-PEG NPs treatment, as shown by the increased integrity of the knee joint (Fig. 7c). Statistically, bone volume (BV), bone volume/total tissue volume (BV/TV), and trabecular number (Tb.N) of subchondral bone was reduced while trabecular separation (Tb.Sp) was increased in ACLT-induced mice compared with the vehicle group. However, PDA-PEG NPs treatment upregulated BV and BV/TV to some extent, meanwhile significantly promoted Tb.N and reduced Tb.Sp in OA mice (Fig. 7d). Microfil perfusion results showed that both volume and number of blood vessels in the subchondral bone were significantly increased in OA mice. However, PDA-PEG NPs suppressed vessel number and volume in subchondral bone, retaining the angiogenesis similar to mice in the control group (Fig. 7e, f). These data demonstrated that PDA-PEG NPs suppressed subchondral bone resorption and blood vessel formation in vivo.

### PDA-PEG NPs attenuate OA progression

**Fig. 8:**
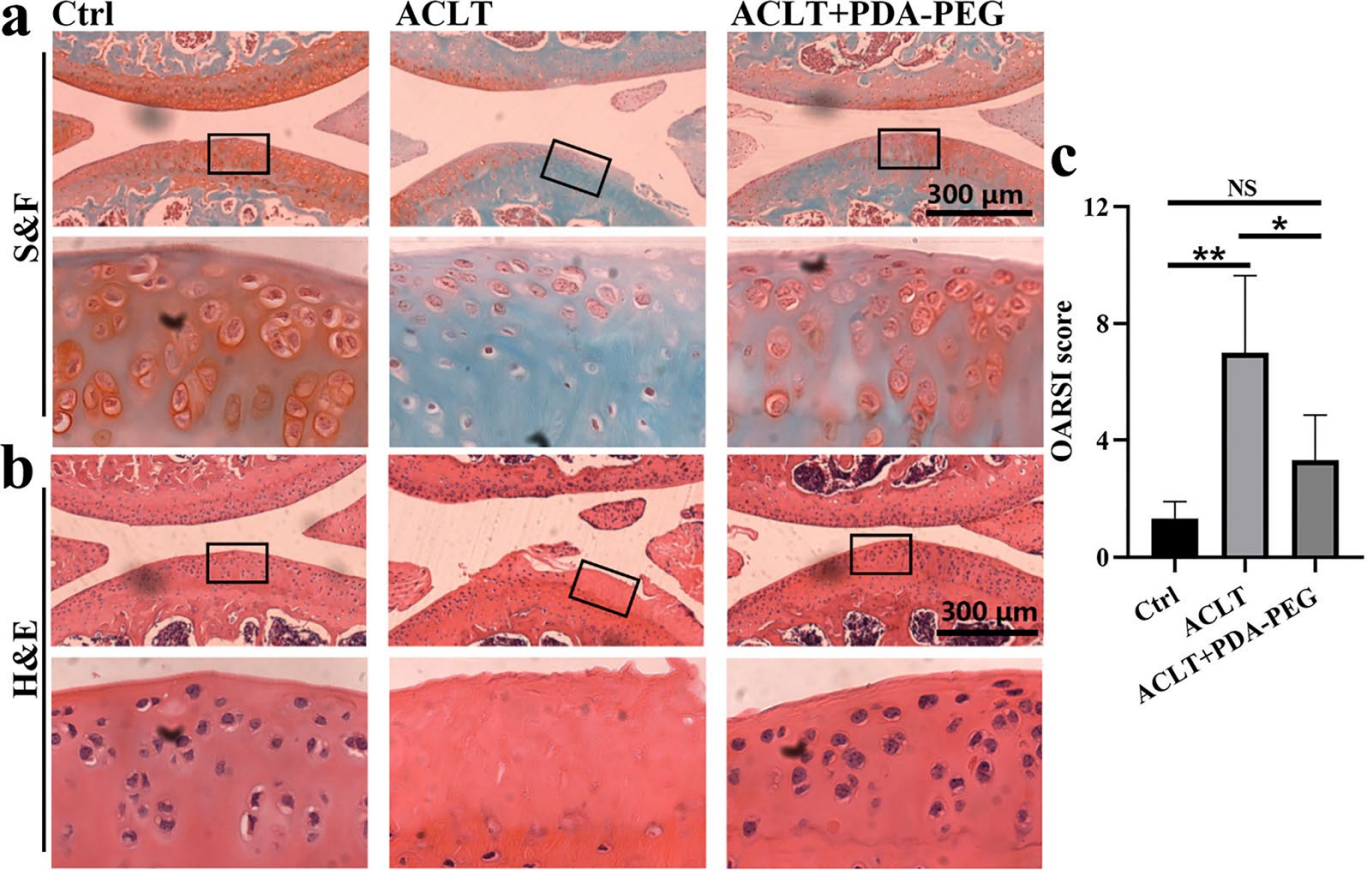
PDA-PEG NPs attenuate cartilage degradation. **a** S&F and **b** H&E staining of the medial compartments of the knee joints, ×50 magnification. **c** OASRI score for assessing the cartilage degradation. n = 3. Symbol “*” denotes p < 0.05, “**” denotes p < 0.01, NS indicates not significant

**Fig. 9:**
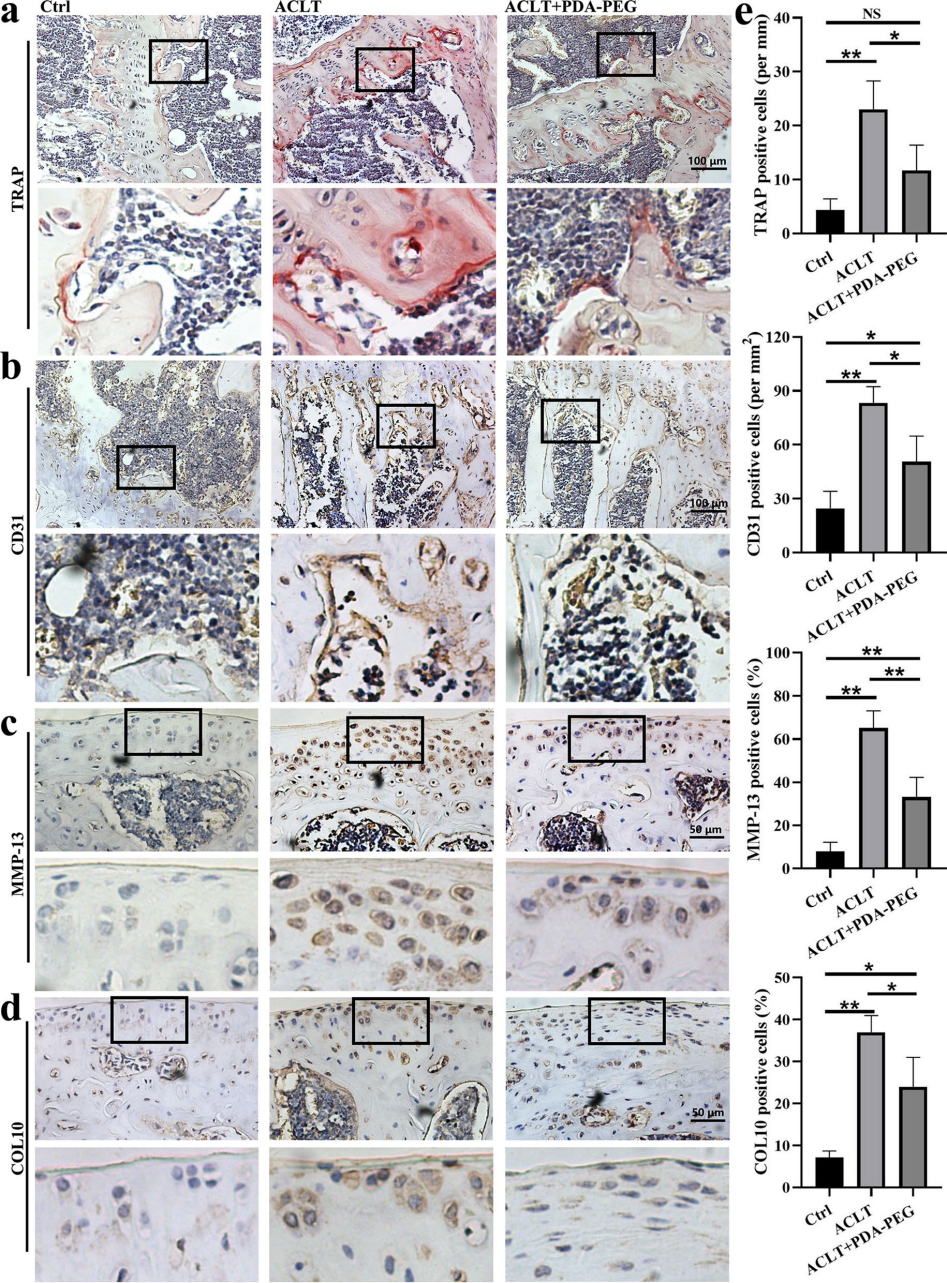
PDA-PEG NPs attenuate OA progression. **a** TRAP and **b** CD31 staining of the subchondral bone, ×200 magnification. **c** MMP-13 and **d** COL10 staining of the cartilage, ×400 magnification. **e** Quantitative analysis TRAP, CD31, MMP-13, and COL10 positively-stained cells. n = 3. Symbol “*” denotes p < 0.05, “**” denotes p < 0.01, NS indicates not significant

In ACLT-induced OA mice, the thickness of articular cartilage was decreased, indicating the loss of proteoglycan. However, PDA-PEG NPs treatment could increase the cartilage integrity and upregulate proteoglycan (Fig. 8a, b). Osteoarthritis Research Society International (OARSI) score of ACLT-induced mice was ~ 7, which was decreased to ~ 3 with the treatment of PDA-PEG NPs (Fig. 8c). Additionally, PDA-PEG NPs inhibited TRAP-positive cells and CD31 expression which respectively indicated formation of osteoclast and blood vessel in the subchondral bone (Fig. 9a, b). Further, expressions of MMP-13 and COL10 in articular cartilage were suppressed by PDA-PEG NPs (Fig. 9c, d). Quantitative analysis indicated that the number of osteoclasts, blood vessel, MMP-3, and COL10-positive cells were distinctly reduced with PDA-PEG NPs treatment (Fig. 9e). These data showed that PDA-PEG NPs attenuated OA progression induced by ACLT.

### Biosafety evaluation of PDA-PEG NPs

For evaluating the biosafety of PDA-PEG NPs, the internal organs of mice were harvested and administrated to hematoxylin & eosin (H&E) staining. Results showed that there was no obvious histologic damage found in PDA-PEG NPs-treated mice (Additional file 1: Fig. S1). Moreover, blood routine analysis indicated that there were no obvious differences between the PDA-PEG NPs-treated mice and the normal mice (Additional file 1: Table S1).

## Discussion

In this study, spherical PDA-PEG NPs were successfully prepared according to previous reports [27, 37]. PDA-PEG NPs could eliminate ROS both in the acellular and intracellular environments. Moreover, PDA-PEG NPs were demonstrated to inhibit osteoclastogenesis by regulating NF-κB/JNK pathway. Further, PDA-PEG NPs suppressed osteoclast-related angiogenesis via regulating PDGF-BB. In vivo, PDA-PEG NPs suppressed bone resorption and angiogenesis in subchondral bone and attenuated cartilage damage in OA mice. Our results indicated that PDA-PEG NPs could be a potential treatment for OA.

Osteochondral unit is the biocomposite composed of articular cartilage, calcified cartilage, subchondral cortical and trabecular bone, which could transfer load of the burden [38]. In the early stage of OA, the osteochondral unit undergoes several alterations both in composition and structure. In particular, metabolic activity of chondrocytes is increased, accompanied by enhanced expression of proinflammatory cytokines and reduced proteoglycan production in articular cartilage [39]. In subchondral bone, the remodeling of the cortical plate is enhanced, leading to increased porosity and reduced bone mass. The subchondral cortical plate thickness is increased, usually accompanied by osteophyte and bone cyst formation [10]. During the evolution of OA, the specific changes in osteochondral unit including composition, function, and structure could be therapeutic targets for OA.

Currently, various treatments including pharmacological and non-pharmacological therapies have been applied to treat OA [40]. However, no effective treatment has been found for OA until the end stage necessitating joint replacement [41]. Recently, several biomaterials including scaffolds or 3D scaffolds have been synthesized, which could regulate microenvironments, promote bone regeneration [42–44]. These biofunctionalized materials provide a new strategy for the treatment of OA. Subchondral bone including subchondral cancellous bone and cortical plate is capable of distributing stress and maintaining conformation of the joint. Subchondral bone deterioration is commonly related to articular cartilage degradation and damage [45]. In the early stage of OA, osteoclast activity was increased, which could cause abnormal subchondral bone remodeling. This might further alter joint shape and load transmission that then predispose to cartilage loss [13]. Our data showed that the bone resorption in subchondral bone was increased in ACLT-induced OA mice, as signified by reduced BV, BV/TV, and Tb.N and increased Tb.Sp in subchondral bone. The deteriorated subchondral bone might account for the cartilage degradation. A previous study has demonstrated that enhancing the integrity of subchondral bone could delay cartilage damage in the progression of OA [46]. Moreover, clinical research found that anti-osteoporotic drugs including bisphosphonates (BPs) could have therapeutic effects on OA [47, 48]. These reports suggested that inhibiting osteoclastogenesis could be a potential therapy for OA. In this study, we systematically administrated PDA-PEG NPs after ACLT operation, the osteoclast activation was inhibited at the beginning of OA, which may be beneficial for protecting the cartilage. The results also confirmed that loss of cartilage was rescued after PDA-PEG NPs administration.

ROS-induced oxidative stress injury, which could lead to cartilage degeneration, is an important destructive factor for OA progression [49]. During osteoclast differentiation induced by RANKL, ROS played a key role in activating several signaling pathways including NF-κB and MAPK signaling [50]. In the NF-κB pathway, phosphorylated IκBα could further promote the phosphorylation of p65 subunit. Phosphorylated p65 could translocate to the nucleus then bind to specific DNA sites, further activating expression of osteoclastogenesis-related genes [51]. In this study, we proved that IκBα was increased with the stimulation of PDA-PEG NPs, which further decreased phosphorylation of p65. MAPK pathway involved in regulating differentiation, proliferation, and innate immunity is also important for osteoclast activation [52, 53]. JNK, ERK, and p38 kinases are critical and also the most studied proteins of MAPK pathway [54]. Our data showed that RANKL promoted expression of p-JNK, p-ERK, and p-p38 compared with BMMs without RANKL stimulation. Moreover, PDA-PEG NPs at 100 µg/ml could inhibit the phosphorylation of JNK while not affecting the phosphorylation of ERK and p38. These data suggested that PDA-PEG NPs might inhibit RANKL-induced osteoclastogenesis by modulating NF-κB/JNK signaling pathway.

The process of osteoclast differentiation is influenced by numerous factors. NFATC1 was reported to be a key transcription factor for osteoclastogenesis [35]. NFATC1 is capable of activating various genes including TRAP, c-FOS, cathepsin K, DC-STAMP, and CALCR, which are important for osteoclast differentiation and function [55]. Our data demonstrated that expression of NFATC1, TRAP, c-FOS, cathepsin K, DC-STAMP, and CALCR were distinctly upregulated with the stimulation of RANKL. However, both NFATC1 and other osteoclast differentiation-related genes were down-regulated with the administration of PDA-PEG NPs in a dose-dependent manner. Likewise, western blotting reconfirmed that PDA-PEG NPs suppressed the expression of NFATC1, signifying that PDA-PEG NPs were effective in inhibiting osteoclast formation.

PDA and its derivatives have been widely employed in biomedical researches due to their high antioxidant activity and biocompatibility [25]. Several studies have demonstrated that PDA NPs were capable of scavenging multiple ROS in vitro and in vivo [29, 56], therefore, PDA NPs were suitable for ROS-induced injury. In this study, the cytotoxicity of PDA-PEG NPs was first evaluated in vitro. Results showed that PDA NPs had low cytotoxicity even at a high concentration. Further, we determined the biosafety of PDA-PEG NPs in vivo. Both H&E staining and blood routine analysis demonstrated that PDA-PEG NPs had good biosecurity. Moreover, the ROS scavenging ability of PDA-PEG NPs was assessed in acellular/intracellular environments. Results confirmed the superior antioxidant activity of PDA-PEG NPs.

Currently, ACLT and destabilization of the medial meniscus (DMM) are the two mostly-used OA models which are induced by surgery. However, remodeling of the subchondral bone in the early stage of OA has been shown only in the ACLT-induced OA model [57, 58]. Herein, we administrated ACLT-induced OA model in this study to better observing the changes in subchondral bone. Our data demonstrated that ACLT indeed caused bone resorption in subchondral bone, while PDA-PEG NPs protected subchondral bone microarchitecture through decreasing osteoclast formation and angiogenesis.

In this study, we deployed PDA-PEG NPs via intraperitoneal injection instead of intra-articular injection. For one thing, we aimed to assess the effects of PDA-PEG NPs on osteoclastogenesis and angiogenesis in subchondral bone. Nevertheless, PDA-PEG NPs may not effectively penetrate subchondral bone via intra-articular injection owing to the obstruction of cartilage, calcified cartilage, and subchondral cortical bone. For another, repeated arthrocentesis, although rare, may cause joint infection [59]. Thus, we applied intraperitoneal injection in this study.

This study has a few limitations as follows. First, OA is a whole-joint disorder as mentioned previously, however, we focused on the subchondral pathological changes including osteoclastogenesis and angiogenesis. This doesn’t imply that other pathological changes such as synovitis and osteophyte formation are not important. As we aimed to assess the effects of PDA-PEG NPs on the crosstalk between cartilage and subchondral bone. Second, analyses on articular joint tissues are limited in this study. In fact, magnetic resonance imaging (MRI) of mice might better visualize the pathological changes in the whole joints. Third, in micro-CT analysis, we focused on bone remodeling in the subchondral bone while ignoring osteophyte formation. Despite these limitations, our study is meaningful in exploring new avenues for treating OA.

## Conclusion

In conclusion, we developed an effective ROS scavenger for OA treatment. PDA-PEG NPs showed low toxicity both in vitro and in vivo. Moreover, PDA-PEG NPs could inhibit osteoclastogenesis and osteoclast-related angiogenesis. Intraperitoneal injection of PDA-PEG NPs rescued subchondral bone deterioration and attenuate cartilage degradation. The therapeutic effects of PDA-PEG NPs were briefly described in graphical abstracts. Although these data suggested that PDA-PEG NPs might have therapeutic potential for OA, further studies from different levels are needed to substantiate the efficacy of PDA-PEG NPs.

## Materials and methods

### Cells, media, and reagents

Dopamine hydrochloride and mPEG-NH_2_ were purchased from Sigma-Aldrich (USA). Primary BMMs were cultured in α-minimum essential medium (α-MEM; HyClone, USA) containing 10% fetal bovine serum (FBS; Gibco, USA), 100 U/ml penicillin, and 100 µg/ml streptomycin (Gibco, USA) at 37 °C, 5% CO_2_. RANKL and M-CSF were purchased from PeproTech (USA). H_2_O_2_ solution was purchased from Beyotime Biotechnology (China) while kept at 4℃ while protected from light.

### Synthesis of PDA-PEG NPs

PDA-PEG NPs were synthesized as previously reported [27, 37]. Briefly, 100 mg dopamine hydrochloride was dissolved in 50 ml deionized of water. Under vigorous stirring, 0.4 ml of 1 M NaOH was added to the above solution. After self-polymerization for 2 h, the as-obtained PDA NPs were collected after centrifugation (14,000 rpm, 10 min) then washed with deionized water three times. PDA-PEG NPs were prepared via mixing PDA NPs with mPEG-NH_2_ at a ratio of 1:2. After stirring for 24 h, the PDA-PEG NPs were purified by centrifugation (14,000 rpm, 10 min) then washed with deionized water three times (Fig. 1a).

### Characterization of PDA-PEG NPs

PDA-PEG NPs were photographed with a TEM (JEOL 200CX, Japan). DLS and zeta potential of PDA-PEG NPs were measured with a Malvern Zetasizer instrument (UK). The elemental concentrations of PDA and PDA-PEG NPs including N, C, and H were measured with an automatic elemental analyzer (Elementar vario EL III, Germany). XPS spectrum was measured with Thermo Scientific K-Alpha (USA). FT-IR spectra of PDA and PDA-PEG NPs have measured a Nicolet iS20 spectrometer (Thermo Scientific, USA). XRD was measured using a SmartLab SE device (Japan) at Cu Kα with a scanning rate of 2 min^− 1^. The UV–Vis-NIR absorption spectra of PDA and PDA-PEG NPs were measured with a UV-3600i Plus device (Japan).

### BMMs isolation and culture

All animal-related procedures were approved by the Ethics Committee of the First Affiliated Hospital of Soochow University. BMMs were harvested from femurs and tibias of 6-week-old C57BL/6J mice referring to a previous study [60]. Briefly, after sacrificing by anesthetic overdose, femurs and tibias of the mice were harvested. Bone marrow was flushed from tibias and femurs then resuspended in total α-MEM medium containing 30 ng/ml M-CSF. The media was changed every other day to remove the non-adherent cells. The BMMs at 80% confluence were washed with PBS and then trypsinized for the following experiments.

### Cytotoxicity assay

The effects of PDA-PEG NPs on cell viability were assessed by CCK-8 (Dojindo, Japan). BMMs (10^4^ cells/well) were seeded in a 96-well plate and cultured for 12 h. Further, the cells were treated with various concentrations of PDA-PEG NPs and H_2_O_2_ at different times. 100 µl of 10% CCK-8 solution was added to each well, then the cells were incubated for 2 h. The absorbance was measured at 450 nm with a microplate reader (Bio-Rad, USA). Cell viability was normalized to the control group.

### ABTS and DPPH assay

The antioxidant activities of PDA-PEG NPs in water and organic solvent were evaluated by ABTS and DPPH assay, respectively. A total antioxidant capacity assay kit with a rapid ABTS method was purchased from Beyotime Biotechnology (China). The green ABTS^•+^ could be catalyzed to form ABTS in the presence of antioxidants. The degree of discolouration was quantified as a drop in the absorbance of 734 nm [61]. The antioxidant activities of PDA-PEG NPs were calculated referring to Trolox, which was used as a standard. DPPH (Nanjing Jiancheng Bioengineering Institute, China) was dissolved in 80% methanol. PDA and PDA-PEG NPs at various concentrations (0, 25, 50, and 100 µg/ml) were mixed with 100 µL DPPH solution and incubated for 30 min in the dark. The absorbance of reaction mixtures was detected at a wavelength of 517 nm. The radical scavenging activity was calculated referring to a previous study [62].

### ESR assay

ESR paramagnetic spectrometer (JEOL-FA200, Japan) was utilized to assess the ability of PDA-PEG NPs to scavenge ROS in an acellular environment. Scavenging of O2·^−^ was evaluated by mixing PDA-PEG NPs (100 µg/ml) with 100 mM 5,5-dimethylpyrroline N-oxide (DMPO; Dojindo, Japan), 0.5 mM hypoxanthine (Solarbio Life Science, China), and 0.05 U/ml xanthine oxidase (Solarbio Life Science, China), then incubated in 100% ethanol solution for 1 min. Then, the content of O2·^−^ was determined using ESR according to the manufacturer’s instructions.

### Intracellular ROS detection

ROS in BMMs were measured by a DCFH-DA probe (Beyotime Biotechnology, China) referring to a previous study [63]. BMMs were cultured with or without 50 ng/ml RANKL for 24 h. During incubation, cells were administrated with different concentrations of PDA-PEG NPs. DCFH-DA solution was added at the concentration of 10 µM, and the cells were incubated in the dark for 20 min. Fluorescent signals were observed by fluorescence microscopy (Leica TCS-SP5, Germany). Further, ROS-positive cells in each field were calculated.

### TRAP and actin ring staining assay

BMMs (10^4^ cells/well) were seeded in a 96-well plate. After 12 h, BMMs were treated with 50 ng/ml RANKL and 30 ng/ml M-CSF for inducing osteoclast differentiation. During the induction, cells were treated with varying concentrations of PDA-PEG NPs (0, 25, 50, and 100 µg/ml). After inducing for 3 or 7 days, the cells were fixed with 4% paraformaldehyde for 30 min. A TRAP staining kit (Sigma-Aldrich, USA) was deployed referring to the manufacturer’s instruction. Osteoclasts were identified as TRAP-positive cells which contained more than three nuclei. The number of TRAP-positive cells in each field were calculated using Image-Pro Plus software (Media Cybernetics, USA). For actin ring staining, BMMs were induced for osteoclast formation containing varying concentrations of PDA-PEG NPs (0, 25, 50, and 100 µg/ml) for 3 days. Then cells were successively stained with rhodamine-conjugated phalloidin (Abcam, USA) for 30 min and 4′,6-diamidino-2-phenylindole (DAPI; Sigma-Aldrich, USA) for 10 min. The actin rings were observed by fluorescence microscopy and calculated with Image-Pro Plus software.

### qRT-PCR assay

Total RNA of cells was extracted from different groups using TRIzol reagent (Invitrogen, USA). cDNA synthesis was performed with TaqMan Reverse Transcription reagent (Applied Biosystems, USA), then the transcription-PCR was performed by real-time PCR (ABI 7500, USA). The primers were shown in Additional file 1: Table S2. Relative mRNA expression was normalized to GAPDH mRNA level and calculated by 2^−ΔΔCt^.

### Western blotting

As reported previously [64], BMMs were washed with PBS and lysed using the radioimmunoprecipitation assay buffer (RIPA; Millipore, USA) containing protease and phosphatase inhibitors (Sigma-Aldrich, USA). The protein concentrations were determined using a BCA protein assay kit (Beyotime Biotechnology, China). Total 20 µg proteins were separated by 10% sodium dodecyl sulfate–polyacrylamide gel electrophoresis (SDS-PAGE) and then transferred to polyvinylidene fluoride (PVDF) membrane. The PVDF membranes were further blocked with 5% non-fat milk for 2 h and incubated with primary antibodies at 4 °C overnight. Finally, PVDF membranes were incubated with secondary antibodies for 1 h then washed with tris-buffered saline with Tween 20 (TBST) for three times. The fluorescent signals were detected using the Odyssey imaging system (Li-Cor, USA). NF-κB family antibodies including p-IκBα, IκBα, p-p65, and p65 were purchased from Cell Signaling Technology (USA) and used at a dilution of 1:1000. MAPK family antibodies including p-JNK, JNK, p-ERK, ERK, p-p38, and p38 were purchased from Cell Signaling Technology (USA) and used at a dilution of 1:1000. NFATC1 and GAPDH antibodies were obtained from Abcam (USA) and applied at a dilution of 1:1000.

### ELISA assessment

BMMs (5 × 10^5^ cells/well) seeded in 6-well plates were stimulated with 30 ng/ml M-CSF, 50 ng/ml RANKL, and various concentrations of PDA-PEG for 3 days. The supernatant of cells was centrifuged (1400 rpm, 5 min) to obtain OC-CM. The concentration of PDGF-BB in OC-CM was measured via a PDGF-BB ELISA kit (Abcam, USA) according to the manufacturer’s guidelines.

### Tube formation assay

For assessing the effects of PDA-PEG NPs on osteoclast-related angiogenesis, the tube formation assay was utilized. The OC-CM was respectively administrated with exogenous recombinant PDGF-BB (Peprotech, USA) at 100 ng/ml, anti-PDGF-BB antibody (Millipore, USA) at 10 µg/ml, or PDA-PEG NPs prepared as mentioned above at 100 µg/ml. According to a previous study [65], 50 µl matrigel (BD Biosciences, USA) was deployed to a 96-well plate, then polymerized 1 h at 37 °C to form a gel-like surface. HUVECs were seeded at 10^4^ cells/well and incubated with various OC-CM for 6 h at 37 °C in 5% CO_2_. Then, HUVECs were stained with calcein-AM then photographed with fluorescence microscopy. The tube length in each field was quantified using Image-Pro Plus software.

### Migration assay

8-µm transwell chambers (Millipore, USA) were utilized to evaluate the migration of HUVECs. The chambers were placed on a 24-well plate, then HUVECs (2 × 10^4^ cells) in 200 µl medium were placed in the chamber, meanwhile, 500 µl medium was added into the plate. Cells were incubated with OC-CM and PDA-PEG NPs at 37℃ for 12 h. Then cells remaining in the upper chamber were scraped with cotton swabs, cells in the lower room were fixed in 4% paraformaldehyde for 30 min and stained with crystal violet for 20 min. The migrated cells were photographed with a light microscope.

### Establishment of ACLT-induced OA mouse model

Eighteen C57BL/6J mice (3-month-old male) were obtained from Shanghai SLAC Laboratory Animal Company (China). Mice were fed in specific pathogen-free (SPF) conditions and allowed free access to commercial food and water. ACLT was deployed to induce abnormal mechanical loading-associated OA of the right knee as described in previous studies [66, 67]. The sham operation was conducted via opening the joint capsule and then suturing the incision. Eighteen mice were evenly and randomly distributed into three groups. Mice in group 1 were deployed to a sham operation, while mice in groups 2 and 3 were subjected to ACLT of the right knee. Furthermore, mice in group 2 were intraperitoneally injected with PBS, meanwhile, mice in group 3 were intraperitoneally injected with PDA-PEG NPs (10 mg/kg) every other day until 4 weeks. PDA-PEG NPs were deployed to mice 3 days after the ACLT induction. The concentration of PDA-PEG NPs used in this study was referred to in a previous study [27]. At the end of the injection, the mice were euthanized by anesthetic overdose.

### Blood routine examination

At the end of the 4 weeks post-injection, 200 µl blood sample from each mouse was harvested from the eyeball then collected in heparinized tubes. The samples were evaluated by Mindray BC-2800vet for analyzing the blood routine indexes.

### Micro-CT scanning

The knee joints of mice were separated then fixed in 4% paraformaldehyde for 48 h. Specimens were scanned with micro-CT (µCT 80; Scanco, Zurich, Switzerland) according to a previous study [68]. The parameters of micro-CT used in this study were as follows: voltage 70 kV, electric current 114 µA, resolution of 10 μm per pixel. After scanning, three-dimensional structural parameters which include BV, BV/TV, Tb.N, and Tb. Sp was analyzed. The region of interest (ROI) was defined as the whole subchondral trabecular bone in tibial plateaus of the knee joints.

### Micro-CT based microangiography

To display blood vessels in subchondral bone, microfilm injection compounds (Microfil MV-130, Flow Tech, USA) for angiography were deployed. Mice were euthanized, then 0.9% normal saline solution containing 100 U/ml heparin sodium was used to flush the vascular system through the left ventricle. Further, 4% paraformaldehyde was injected to fix the mice through the left ventricle as well. The freshly prepared microfil mixture was pressured through the left ventricle. Mice were placed at 4 °C overnight to ensure contrast agent polymerization, then mice were dissected to harvest the knee joints. Further, the joints were fixed with 4% paraformaldehyde for 48 h. Specimens were decalcified in 10% EDTA for another 48 h to facilitate density differences of the vasculature from the surrounding tissues [18]. Specimens were scanned by micro-CT at a resolution of 9 μm isotropic voxel size, and a threshold of 180 based on the two-dimensional tomograms. Vessel volume (VV) and vessel number (VN) in the subchondral bone were analyzed.

### Histological observation

Samples were decalcified in 10% EDTA for 4 weeks, then embedded with paraffin. Sagittal sections were cut at 4 μm of the medial compartment of the knee joint and deployed to H&E and Safranin O-fast green (S&F) staining. OARSI score was calculated to evaluate cartilage damage as previously described [69]. The OARSI scoring was based on grade (0–6) and stage (0–4). OARSI score = grade × stage, and OARSI scores range from 0 to 24 using this calculation method. To assess the biosafety of PDA-PEG NPs, internal organs including heart, liver, spleen, lung, and kidney were embedded with paraffin for sectioning and H&E staining. Immunohistochemical staining was deployed to antibodies against TRAP, CD31, MMP-13, and COL10 (Abcam, USA) at a dilution of 1:200. Sections were incubated with diaminobenzene then counterstained with hematoxylin. The number of positively stained cells was counted in per section. The proportion of positively stained cells was calculated. Three samples in each group and three sequential sections in each sample were stained and assessed. Images were photographed with a light microscope.

### Statistical analysis

Data were expressed as mean ± standard deviation. Student’s t test was applied for comparisons between two groups. One-way analysis of variance (ANOVA) was used for comparisons among three or more groups. P < 0.05 was considered significantly different. The symbol “*” denoted p < 0.05, and “**” denoted p < 0.01. All data analysis was conducted with SPSS 22.0 analysis software (SPSS Inc, Chicago, IL, USA).

## Supplementary Information

**Additional file 1: Figure S1.** H&E staining of heart, liver, spleen, lung, and kidney after PDA-PEG NPs treatment. **Table S1.** Blood analysis of mice treated with or without PDA-PEG NPs. n = 6. **Table S2.** Primer sequences used in the article.

## Data Availability

Most of the generated data analysis and results in this study are included in this published article.

## References

[1] Hügle T, Geurts J (2017). What drives osteoarthritis?-synovial versus subchondral bone pathology. Rheumatology (Oxford).

[2] Findlay DM, Kuliwaba JS (2016). Bone-cartilage crosstalk: a conversation for understanding osteoarthritis. Bone Res.

[3] Daugaard R, Tjur M, Sliepen M (2018). Are patients with knee osteoarthritis and patients with knee joint replacement as physically active as healthy persons?. J Orthop Translat.

[4] Longo UG, Loppini M, Fumo C (2012). Osteoarthritis: new insights in animal models. Open Orthop J.

[5] McAlindon TE, Bannuru RR, Sullivan MC (2014). OARSI guidelines for the non-surgical management of knee osteoarthritis. Osteoarthritis Cartilage.

[6] Melcarne L, Garcia-Iglesias P, Calvet X (2016). Management of NSAID-associated peptic ulcer disease. Expert Rev Gastroenterol Hepatol.

[7] Bijlsma JW, Berenbaum F, Lafeber FP (2011). Osteoarthritis: an update with relevance for clinical practice. Lancet.

[8] Zhu X, Chen F, Lu K (2019). PPARγ preservation via promoter demethylation alleviates osteoarthritis in mice. Ann Rheum Dis.

[9] Kapoor M, Martel-Pelletier J, Lajeunesse D, Pelletier JP, Fahmi H (2011). Role of proinflammatory cytokines in the pathophysiology of osteoarthritis. Nat Rev Rheumatol.

[10] Goldring SR, Goldring MB (2016). Changes in the osteochondral unit during osteoarthritis: structure, function and cartilage-bone crosstalk. Nat Rev Rheumatol.

[11] Mu W, Xu B, Ma H (2018). Halofuginone attenuates osteoarthritis by rescuing bone remodeling in subchondral bone through oral gavage. Front Pharmacol.

[12] Zhao C, Liu Q, Wang K (2017). Artesunate attenuates ACLT-induced osteoarthritis by suppressing osteoclastogenesis and aberrant angiogenesis. Biomed Pharmacother.

[13] Burr DB, Gallant MA (2012). Bone remodelling in osteoarthritis. Nat Rev Rheumatol.

[14] Siebelt M, Waarsing JH, Groen HC (2014). Inhibited osteoclastic bone resorption through alendronate treatment in rats reduces severe osteoarthritis progression. Bone.

[15] Mapp PI, Walsh DA, Bowyer J, Maciewicz RA (2010). Effects of a metalloproteinase inhibitor on osteochondral angiogenesis, chondropathy and pain behavior in a rat model of osteoarthritis. Osteoarthr Cartil.

[16] Han Y, You X, Xing W, Zhang Z, Zou W (2018). Paracrine and endocrine actions of bone-the functions of secretory proteins from osteoblasts, osteocytes, and osteoclasts. Bone Res.

[17] Xie H, Cui Z, Wang L (2014). PDGF-BB secreted by preosteoclasts induces angiogenesis during coupling with osteogenesis. Nat Med.

[18] Cui Z, Crane J, Xie H (2016). Halofuginone attenuates osteoarthritis by inhibition of TGF-beta activity and H-type vessel formation in subchondral bone. Ann Rheum Dis.

[19] Shen C, Cai GQ, Peng JP, Chen XD (2015). Autophagy protects chondrocytes from glucocorticoids-induced apoptosis via ROS/Akt/FOXO3 signaling. Osteoarthritis Cartilage.

[20] Finkel T (2012). Signal transduction by mitochondrial oxidants. J Biol Chem.

[21] Finkel T (2011). Signal transduction by reactive oxygen species. J Cell Biol.

[22] Zhou F, Mei J, Yuan K (2019). Isorhamnetin attenuates osteoarthritis by inhibiting osteoclastogenesis and protecting chondrocytes through modulating reactive oxygen species homeostasis. J Cell Mol Med.

[23] Wang T, Fan Q, Hong J (2021). Therapeutic nanoparticles from grape seed for modulating oxidative stress. Small.

[24] Yang P, Zhu F, Zhang Z (2021). Stimuli-responsive polydopamine-based smart materials. Chem Soc Rev.

[25] Liu Y, Ai K, Lu L (2014). Polydopamine and its derivative materials: synthesis and promising applications in energy, environmental, and biomedical fields. Chem Rev.

[26] Wu D, Zhou J, Creyer MN (2021). Phenolic-enabled nanotechnology: versatile particle engineering for biomedicine. Chem Soc Rev.

[27] Zhao H, Zeng Z, Liu L (2018). Polydopamine nanoparticles for the treatment of acute inflammation-induced injury. Nanoscale.

[28] Yang P, Gu Z, Zhu F, Li Y (2020). Structural and functional tailoring of melanin-like polydopamine radical scavengers. CCS Chem.

[29] Bao X, Zhao J, Sun J, Hu M, Yang X (2018). Polydopamine nanoparticles as efficient scavengers for reactive oxygen species in periodontal disease. ACS Nano.

[30] Zhou J, Lin Z, Ju Y (2020). Polyphenol-mediated assembly for particle engineering. Acc Chem Res.

[31] Gao N, Xing C, Wang H (2019). pH-Responsive dual drug-loaded nanocarriers based on poly (2-ethyl-2-oxazoline) modified black phosphorus nanosheets for cancer chemo/photothermal therapy. Front Pharmacol.

[32] Luo H, Gu C, Zheng W (2015). Facile synthesis of novel size-controlled antibacterial hybrid spheres using silver nanoparticles loaded with poly-dopamine spheres. RSC Adv.

[33] Dong Z, Gong H, Gao M (2016). Polydopamine nanoparticles as a versatile molecular loading platform to enable imaging-guided cancer combination therapy. Theranostics.

[34] Boyle WJ, Simonet WS, Lacey DL (2003). Osteoclast differentiation and activation. Nature.

[35] Kim K, Kim JH, Youn BU, Jin HM, Kim N (2010). Pim-1 regulates RANKL-induced osteoclastogenesis via NF-kappaB activation and NFATc1 induction. J Immunol.

[36] Hu Z, Chen Y, Song L (2018). Flavopiridol protects bone tissue by attenuating RANKL induced osteoclast formation. Front Pharmacol.

[37] Chen Y, Zhang F, Wang Q (2018). The synthesis of LA-Fe3O4@PDA-PEG-DOX for photothermal therapy-chemotherapy. Dalton Trans.

[38] Molfetta L, Casabella A, Rosini S, Saviola G, Palermo A (2022). Role of the osteochondral unit in the pathogenesis of osteoarthritis: focus on the potential use of clodronate. Curr Rheumatol Rev.

[39] Zheng L, Zhang Z, Sheng P, Mobasheri A (2021). The role of metabolism in chondrocyte dysfunction and the progression of osteoarthritis. Ageing Res Rev.

[40] Hochberg MC, Altman RD, April KT (2012). American College of Rheumatology 2012 recommendations for the use of nonpharmacologic and pharmacologic therapies in osteoarthritis of the hand, hip, and knee. Arthritis Care Res (Hoboken).

[41] Hawker GA, Mian S, Bednis K, Stanaitis I (2011). Osteoarthritis year 2010 in review: non-pharmacologic therapy. Osteoarthr Cartil.

[42] Zhu T, Cui Y, Zhang M (2020). Engineered three-dimensional scaffolds for enhanced bone regeneration in osteonecrosis. Bioact Mater.

[43] Zhao D, Zhu T, Li J (2021). Poly(lactic-co-glycolic acid)-based composite bone-substitute materials. Bioact Mater.

[44] Zhu T, Jiang M, Zhang M (2022). Biofunctionalized composite scaffold to potentiate osteoconduction, angiogenesis, and favorable metabolic microenvironment for osteonecrosis therapy. Bioact Mater.

[45] Madry H, van Dijk CN, Mueller-Gerbl M (2010). The basic science of the subchondral bone. Knee Surg Sports Traumatol Arthrosc.

[46] Bellido M, Lugo L, Roman-Blas JA (2011). Improving subchondral bone integrity reduces progression of cartilage damage in experimental osteoarthritis preceded by osteoporosis. Osteoarthr Cartil.

[47] Herrero-Beaumont G, Roman-Blas JA, Osteoarthritis (2013). Osteoporotic OA: a reasonable target for bone-acting agents. Nat Rev Rheumatol.

[48] Lems WF (2018). Bisphosphonates: a therapeutic option for knee osteoarthritis?. Ann Rheum Dis.

[49] Reed KN, Wilson G, Pearsall A, Grishko VI (2014). The role of mitochondrial reactive oxygen species in cartilage matrix destruction. Mol Cell Biochem.

[50] Lee NK, Choi YG, Baik JY (2005). A crucial role for reactive oxygen species in RANKL-induced osteoclast differentiation. Blood.

[51] Abu-Amer Y (2013). NF-kappaB signaling and bone resorption. Osteoporos Int.

[52] Kong X, Yang Y, Wu W (2015). Triterpenoid saponin W3 from anemone flaccida suppresses osteoclast differentiation through inhibiting activation of MAPKs and NF-kappaB pathways. Int J Biol Sci.

[53] Tao H, Okamoto M, Nishikawa M, Yoshikawa H, Myoui A (2011). P38 mitogen-activated protein kinase inhibitor, FR167653, inhibits parathyroid hormone related protein-induced osteoclastogenesis and bone resorption. PLoS ONE.

[54] Lee W, Ko KR, Kim HK (2018). Dehydrodiconiferyl alcohol inhibits osteoclast differentiation and ovariectomy-induced bone loss through acting as an estrogen receptor agonist. J Nat Prod.

[55] Song I, Kim JH, Kim K (2009). Regulatory mechanism of NFATc1 in RANKL-induced osteoclast activation. FEBS Lett.

[56] Jodko-Piorecka K, Litwinienko G (2015). Antioxidant activity of dopamine and L-DOPA in lipid micelles and their cooperation with an analogue of alpha-tocopherol. Free Radic Biol Med.

[57] Sniekers YH, Intema F, Lafeber FP (2008). A role for subchondral bone changes in the process of osteoarthritis; a micro-CT study of two canine models. BMC Musculoskelet Disord.

[58] Glasson SS, Blanchet TJ, Morris EA (2007). The surgical destabilization of the medial meniscus (DMM) model of osteoarthritis in the 129/SvEv mouse. Osteoarthritis Cartilage.

[59] Shanmugasundaram S, Vaish A, Chavada V, Murrell WD, Vaishya R (2021). Assessment of safety and efficacy of intra-articular injection of stromal vascular fraction for the treatment of knee osteoarthritis-a systematic review. Int Orthop.

[60] Zheng Z, Zhang X, Huang B (2021). Site-1 protease controls osteoclastogenesis by mediating LC3 transcription. Cell Death Differ.

[61] Munteanu IG, Apetrei C (2021). Analytical methods used in determining antioxidant activity: a review. Int J Mol Sci.

[62] Hemlata GS, Tejavath KK (2021). ROS-mediated apoptosis induced by BSA nanospheres encapsulated with fruit extract of cucumis prophetarum in various human cancer cell lines. ACS Omega.

[63] Liu M, Zhong S, Kong R (2017). Paeonol alleviates interleukin-1beta-induced inflammatory responses in chondrocytes during osteoarthritis. Biomed Pharmacother.

[64] Han J, Gao W, Su D, Liu Y (2018). Gypenoside inhibits RANKL-induced osteoclastogenesis by regulating NF-kappaB, AKT, and MAPK signaling pathways. J Cell Biochem.

[65] Wang TY, Wang W, Li FF (2020). Maggot excretions/secretions promote diabetic wound angiogenesis via miR18a/19a -TSP-1 axis. Diabetes Res Clin Pract.

[66] Lorenz J, Grassel S (2014). Experimental osteoarthritis models in mice. Methods Mol Biol.

[67] Hayami T, Zhuo Y, Wesolowski GA, Pickarski M, Duong LT (2012). Inhibition of cathepsin K reduces cartilage degeneration in the anterior cruciate ligament transection rabbit and murine models of osteoarthritis. Bone.

[68] Qiao H, Wang TY, Yu ZF (2016). Structural simulation of adenosine phosphate via plumbagin and zoledronic acid competitively targets JNK/Erk to synergistically attenuate osteoclastogenesis in a breast cancer model. Cell Death Dis.

[69] Pritzker KP, Gay S, Jimenez SA (2006). Osteoarthritis cartilage histopathology: grading and staging. Osteoarthr Cartil.

