# Supplementary material for: Antioxidant PDA-PEG nanoparticles alleviate early osteoarthritis by inhibiting osteoclastogenesis and angiogenesis in subchondral bone
Source: J Nanobiotechnology. 2022 Nov 16;20:479. doi: 10.1186/s12951-022-01697-y (PMC9670483; doi:10.1186/s12951-022-01697-y)
Supplement: Supplementary file 1 — Additional file 1: Figure S1. H&E staining of heart, liver, spleen, lung, and kidney after PDA-PEG NPs treatment. Table S1. Blood analysis of mice treated with or without PDA-PEG NPs. n = 6. Table S2. Primer sequences used in the article. [file 12951_2022_1697_MOESM1_ESM.docx]

**Antioxidant PDA-PEG nanoparticles alleviate early osteoarthritis by inhibiting osteoclastogenesis and angiogenesis in subchondral bone**

Zhikai Wu^1,2†^, Kai Yuan^3†^, Qian Zhang^1^, Jiong Jiong Guo^1*^, Huilin Yang^1*^, Feng Zhou^1,2*^

^1^ Department of Orthopaedic Surgery, The First Affiliated Hospital of Soochow University, Suzhou, Jiangsu, China.

^2^ Orthopaedic Institute, Soochow University, Suzhou, Jiangsu, China.

^3^ Shanghai Key Laboratory of Orthopaedic Implants, Department of Orthopaedic Surgery, Shanghai Ninth People's Hospital, Shanghai Jiao Tong University School of Medicine, Shanghai, China.

† Zhikai Wu and Kai Yuan contributed equally to this work.

* Corresponding authors:

Jiong Jiong Guo: Department of Orthopaedic Surgery, The First Affiliated Hospital of Soochow University. No. 899 Ping Hai Road, Suzhou, Jiangsu, China. E-mail: drjjguo@163.com.

Huilin Yang: Department of Orthopaedic Surgery, The First Affiliated Hospital of Soochow University. No. 899 Ping Hai Road, Suzhou, Jiangsu, China. E-mail: suzhouspine@163.com.

Feng Zhou: Department of Orthopaedic Surgery, The First Affiliated Hospital of Soochow University. No. 899 Ping Hai Road, Suzhou, Jiangsu, China. E-mail: sdfyyzhoufeng@163.com.


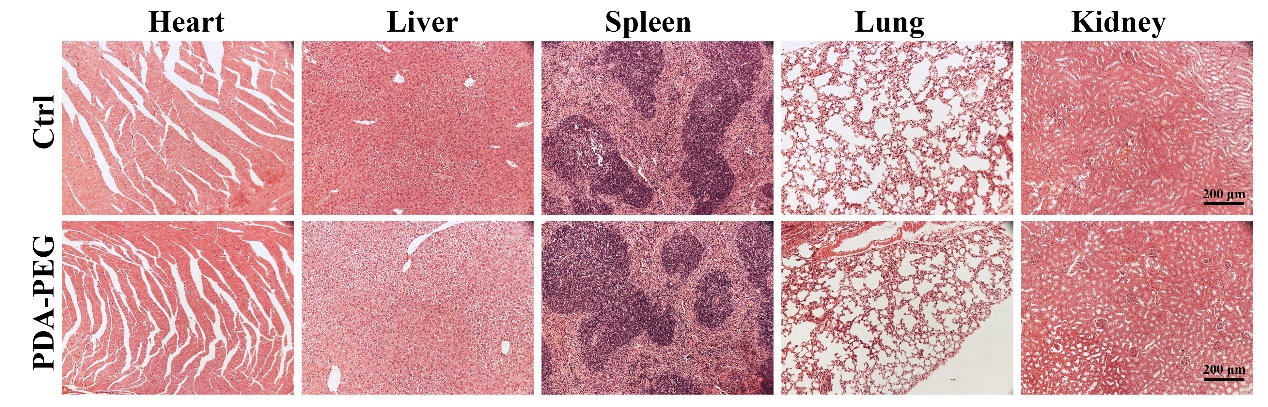


**Figure S1.** H&E staining of heart, liver, spleen, lung, and kidney after PDA-PEG NPs treatment.

**Table S1.** Blood analysis of mice treat with or without PDA-PEG NPs. n=6.

|  | Ctrl | PDA-PEG | p value (t test) |
| --- | --- | --- | --- |
| WBC (10^9^/L) | 6.75±2.21 | 7.07±1.73 | 0.788 |
| Lymph (10^9^/L) | 4.52±1.53 | 5.12±1.55 | 0.515 |
| Mon (10^9^/L) | 0.37±0.31 | 0.30±0.39 | 0.751 |
| Gran (10^9^/L) | 1.87±1.17 | 1.65±1.32 | 0.769 |
| Lymph (%) | 68.58±13.91 | 73.90±17.84 | 0.578 |
| Mon (%) | 5.12±3.53 | 4.37±4.43 | 0.752 |
| Gran (%) | 26.30±10.91 | 21.73±13.49 | 0.533 |
| RBC (10^12^/L) | 9.66±1.16 | 9.79±0.72 | 0.822 |
| HGB (g/L) | 150.83±18.17 | 149.17±9.11 | 0.845 |
| HCT (%) | 46.50±6.54 | 47.00±3.47 | 0.872 |
| MCV (fl) | 48.10±1.21 | 48.07±0.83 | 0.957 |
| MCH (pg) | 15.57±0.19 | 15.22±0.58 | 0.192 |
| MCHC (g/L) | 324.67±9.16 | 317.17±9.02 | 0.183 |
| RDW (%) | 13.85±0.36 | 14.20±0.41 | 0.146 |
| PLT (10^9^/L) | 858.17±254.06 | 996.67±461.51 | 0.534 |
| MPV (fl) | 7.20±0.52 | 7.05±0.24 | 0.537 |
| PDW | 16.83±0.30 | 16.93±0.50 | 0.682 |
| PCT (%) | 0.54±0.16 | 0.46±0.12 | 0.823 |

**Table S2.** Primer sequences used in the article**.**

| GAPDH-F | 5’-ACCCAGAAGACTGTGGATGG-3’ |
| --- | --- |
| GAPDH-R | 5’-CACATTGGGGGTAGGAACAC-3’ |
| NFATC1-F | 5’-CCGTTGCTTCCAGAAAATAACA-3’ |
| NFATC1-R | 5’-TGTGGGATGTGAACTCGGAA-3’ |
| TRAP-F | 5’-CTGGAGTGCACGATGCCAGCGACA-3’ |
| TRAP-R | 5’-TCCGTGCTCGGCGATGGACCAGA-3’ |
| c-Fos-F | 5’-GTTCGTGAAACACACCAGGC-3’ |
| c-Fos-R | 5’-GGCCTTGACTCACATGCTCT-3’ |
| DC-STAMP-F | 5’-AAAACCCTTGGGCTGTTCTT-3’ |
| DC-STAMP-R | 5’-AATCATGGACGACTCCTTGG-3’ |
| Cathepsin K-F | 5’-TCCGCAATCCTTACCGAATA-3’ |
| Cathepsin K-R | 5’-AACTTGAACACCCACATCCTG-3’ |
| CALCR-F | 5’-GCAACGCTTTCACTTCTGAGA-3’ |
| CALCR-R | 5’-GTTCCCACTGCATTGTCCACA-3’ |
| PDGF-BB-F | 5’-CATCCGCTCCTTTGATGATCTT-3’ |
| PDGF-BB-R | 5’-GTGCTCGGGTCATGTTCAAGT-3’ |
| TGF-β-F | 5’-CTCCCGTGGCTTCTAGTGC-3’ |
| TGF-β-R | 5’-GCCTTAGTTTGGACAGGATCTG-3’ |
| VEGFA-F | 5’-CTGCCGTCCGATTGAGACC-3’ |
| VEGFA-R | 5’-CCCCTCCTTGTACCACTGTC-3’ |
| Ang-F | 5’-CCAGGCCCGTTGTTCTTGAT-3’ |
| Ang-R | 5’-GGAAGGGAGACTTGCTCATTC-3’ |
